# Supplementary material for: Systematic Investigation of Plant-Parasitic Nematodes Associated with Main Subtropical Crops in Guangxi Province, China
Source: Life (Basel). 2021 Nov 3;11(11):1177. doi: 10.3390/life11111177 (PMC8621776; doi:10.3390/life11111177)
Supplement: Supplementary file 1 [file life-11-01177-s001.zip › life-1375527-supplementary.pdf]

life

MDPI

Supplementary data of:

Systematic Investigation of Plant-Parasitic Nematodes Associated with Main Subtropical Crops in Guangxi Province, China

Yi-Xue Mo, Ai-Su Mo, Zhuo-Qiu Qiu, Bing-Xue Li and Hai-Yan Wu *

Guangxi Key Laboratory of Agric-Environment and Agric-Products Safety, Agricultural College of Guangxi University, Nanning 530004, China; 2017304017@st.gxu.edu.cn (Y.-X.M.); winter500@126.com (A.-S.M.); athos0519@163.com (Z.-Q.Q.); 18437958381@163.com (B.-X.L.)

* Correspondence: wuhy@gxu.edu.cn

Table S1. Proportion of major plant parasitic nematodes in different ecological regions.

Ecological Regions	Cities	Genus of Plant Parasitic Nematode				
		Meloidogyne	*Pratylenchus*	*Helicotylenchus*	*Hirschmanniella*	*Tylenchorhynchus*
Central eco-region	Chongzuo	5.71	31.43	34.29	20.00	45.71
	Laibin	13.64	38.64	22.73	38.64	47.73
	Nanning	17.86	19.64	17.86	44.64	26.79
Eastern eco-region	Guigang	24.00	24.00	16.00	32.00	52.00
	Hezhou	0.00	26.09	17.39	34.78	4.35
	Wuzhou	22.58	32.26	19.35	32.26	41.94
	Yulin	17.39	8.60	13.04	13.04	43.48
Northern eco-region	Hechi	12.82	33.33	28.21	46.15	33.33
	Guilin	6.38	10.64	10.64	68.09	36.17
	Liuzhou	5.88	32.35	20.59	50.00	64.71
Southern eco-region	Beihai	14.29	0.00	28.57	28.57	85.71
	Fangchenggang	8.33	41.67	33.33	33.33	58.33
	Qinzhou	0.00	0.00	20.00	16.67	33.33
Western eco-region	Baise	8.33	29.17	45.83	20.83	12.50
